# Irradiation causes senescence, ATP release, and P2X7 receptor isoform switch in glioblastoma

**DOI:** 10.1038/s41419-022-04526-0

**Published:** 2022-01-24

**Authors:** Michele Zanoni, Alba Clara Sarti, Alice Zamagni, Michela Cortesi, Sara Pignatta, Chiara Arienti, Michela Tebaldi, Anna Sarnelli, Antonino Romeo, Daniela Bartolini, Luigino Tosatto, Elena Adinolfi, Anna Tesei, Francesco Di Virgilio

**Affiliations:** 1 Biosciences Laboratory, IRCCS Istituto Romagnolo per lo Studio dei Tumori (IRST) “Dino Amadori”, Meldola, Italy; 2 Department of Medical Sciences, Section of Experimental Medicine, University of Ferrara, Ferrara, Italy; 3 Biostatistics and Clinical Trials Unit, IRCCS Istituto Romagnolo per lo Studio dei Tumori (IRST) “Dino Amadori”, Meldola, Italy; 4 Medical Physics Unit, IRCCS Istituto Romagnolo per lo Studio dei Tumori (IRST) “Dino Amadori”, Meldola, Italy; 5 Radiotherapy Unit, IRCCS Istituto Romagnolo per lo Studio dei Tumori (IRST) “Dino Amadori”, Meldola, Italy; 6 Pathology Unit, M. Bufalini Hospital, AUSL Romagna, Cesena, Italy; 7 Department of Neurosurgery, M. Bufalini Hospital, AUSL Romagna, Cesena, Italy

**Keywords:** Senescence, CNS cancer

## Abstract

Glioblastoma (GBM) is the most lethal brain tumor in adults. Radiation, together with temozolomide is the standard treatment, but nevertheless, relapse occurs in nearly all cases. Understanding the mechanisms underlying radiation resistance may help to find more effective therapies. After radiation treatment, ATP is released into the tumor microenvironment where it binds and activates purinergic P2 receptors, mainly of the P2X7 subtype. Two main P2X7 splice variants, P2X7A and P2X7B, are expressed in most cell types, where they associate with distinct biochemical and functional responses. GBM cells widely differ for the level of P2X7 isoform expression and accordingly for sensitivity to stimulation with extracellular ATP (eATP). Irradiation causes a dramatic shift in P2X7 isoform expression, with the P2X7A isoform being down- and the P2X7B isoform up-modulated, as well as extensive cell death and overexpression of stemness and senescence markers. Treatment with P2X7 blockers during the post-irradiation recovery potentiated irradiation-dependent cytotoxicity, suggesting that P2X7B activation by eATP generated a trophic/growth-promoting stimulus. Altogether, these data show that P2X7A and B receptor isoform levels are inversely modulated during the post-irradiation recovery phase in GBM cells.

## Introduction

Glioblastoma (GBM) is the most aggressive primary brain tumor [1], characterized by a very poor prognosis and a median overall survival of 14.6 months [2]. Current standard treatment consists of surgical resection followed by a combination of radiotherapy and chemotherapy with temozolomide (TMZ) [2, 3]. Despite an initial response to treatment, relapse occurs in almost all cases with a median time to progression of 6.9 months [2, 4]. Treatment failure reflects GBM complexity due to high intra- and inter-tumor heterogeneity [5, 6], a complex microenvironment landscape [7], and difficult drug delivery across the blood–brain barrier. Despite recent advances in understanding phenotypic and genomic features of GBM, no new and more efficient therapies have been introduced during the past several years, thus emphasizing the need for the identification of novel therapeutic targets.

Fundamental pathophysiological processes such as tissue homeostasis, inflammation, and cancer are modulated by purinergic signaling [8]. Key mediators in purinergic signaling are adenine nucleosides (adenosine) and nucleotides (ADP and ATP), acting at P1 and P2 receptors, respectively [9]. ATP, the key energy currency as well as a ubiquitous extracellular messenger and a prototypical DAMP (damage-associated molecular pattern) [10], is released into the extracellular milieu in response to cell stress or injury, and to chemo- and radiotherapy [11, 12]. Effects of extracellular ATP (eATP) in the tumor microenvironment (TME) depend on the repertoire of P2 receptors (P2Rs) and ATP-degrading enzymes (ectonucleotidases) expressed by tumor/stromal/immune cells [13–15]. Among eATP-activated P2Rs, the P2X7 receptor (P2X7Rs) has been extensively implicated in the pathogenesis of several cancer types [13]. This receptor has unique properties and, unlike the other six members of the P2XR family, requires hundreds or even millimolar concentrations of eATP for activation [16]. P2X7R response to eATP is enigmatic since this is a bifunctional receptor, behaving as a Na^+^, K^+^, and Ca^2+^-selective channel and at the same time as a nonselective pore (macropore) permeable to large water-soluble molecules (<900 kDa) [17–20]. Since the P2X7R is non-inactivating, the only means to stop signaling and close the channel/macropore is the hydrolysis of eATP. This explains why, depending on whether undergoes tonic, low-level, activation, or prolonged opening, the P2X7R has dramatically different effects on cell physiology: in the first case, it provides growth-promoting stimulation, while in the latter triggers cell death. This two-faced behavior has important implications in cancer as the P2X7R may on the one hand support tumor progression and on the other trigger an antitumor cytotoxic effect.

The formation of the cytotoxic macropore is regulated by the long intracellular C-terminal tail of the P2X7R [21] and critically modulated by the lipid composition of the plasma membrane [22]. During evolution, and especially in cancer cells, to maximize P2X7R-mediated trophic advantages and mitigate the dire effects caused by uncontrolled macropore-opening, single nucleotide polymorphisms (SNPs), and splicing variants of the *P2RX7* gene originated. Ten human splice variants, four truncated in the C-terminal region, are currently known [23, 24]. One of these, the P2X7B isoform, has attracted hot interest since it retains the ion channel activity but lacks the ability to form the cytotoxic macropore [25]. In cancer, P2X7B shares with the full-length isoform (referred to as P2X7A) the ability to promote tumor growth [26, 27], metastatic dissemination [28, 29], resistance to therapy [30], and invasiveness [31].

The role of the P2X7R in GBM biology is poorly known since contrasting data are reported in the literature. P2X7R stimulation was shown to increase cell proliferation, migration, and release of neoangiogenic and proinflammatory factors in vitro [32, 33]. On the other hand, P2X7R inhibition through silencing increases in vitro and in vivo GL261 murine glioma cells growth [34]. Data from in vivo P2X7R pharmacological blockade are even more contradictory since both growth reduction and increase were reported in the same GBM model [35, 36].

Radiotherapy is the main stake in GBM treatment but unfortunately, resistance to radiation eventually occurs in all patients. Therefore, identification of factors that may increase GBM sensitivity to irradiation (or delay the onset of resistance) would be crucial. The P2X7R has been shown to be a good prognostic indicator of radiosensitivity and a favorable survival marker in GBM patients [37]. However, the mechanism by which the P2X7R enhances sensitivity to irradiation is unknown. In the present study, we investigated the expression and function of P2X7R isoforms A and B in patient-derived GBM cell lines exposed to irradiation.

## Materials and methods

### Cell culture

Patient-derived samples were collected according to a protocol approved by the Italian Local Ethics Committee (CEROM IRST IRCCS-AVR, protocol code: IRST B004) and all the patients have signed informed consent prior to surgery. Primary tumor cells were obtained from patient tumor specimens classified as IV grade GBM by pathologists. Patients’ information were reported in Supplementary Table S1. Briefly collected tumor tissues were washed and enzymatically dissociated. Cells were then plated in NeuroCult NS-A medium (StemCell Technologies, cat n. 05751) supplemented with 20 ng/ml of epidermal- and 10 ng/ml of fibroblast-growth factor (Sigma-Aldrich, cat n. E9644-.2MG and F0291-25UG respectively), 1% penicillin/streptomycin (Gibco^™^, Thermo Fisher Scientific, cat n. 15140-122), and 2% amphotericin B (Euroclone, cat n. ECM0009D) and maintained under hypoxic conditions (1% O_2_) in a humidified 37 °C, 5% CO_2_ incubator. All the primary GBM cell lines were checked periodically for mycoplasma contamination using the MycoAlert^TM^ Mycoplasma Detection Kit (Lonza, cat n. LT07-710) and used between passages 15 and 25 from isolation.

To assess multipotency, GBM cells were differentiated as previously described [38]. Briefly, 3 × 10^4^/cm^2^ GBM cells were plated onto Matrigel-coated glass coverslips in the presence of 20 ng/mL FGF-2. After 2 days, GBM cell cultures were shifted to a mitogen-free medium containing 2% fetal bovine serum (Euroclone, cat n. ECS0180D) for 5 days. Finally, immunofluorescence for astroglial (GFAP) and oligodendroglial (GalC) markers were performed.

### Irradiation treatment

GBM cells were plated at a density of 70–80% and irradiated with 2 Gy, 5 Gy, 7.5 Gy or 10 Gy doses using the linear acceleration Elekta Synergy Platform system (Elekta Oncology Systems, Stockholm, Sweden) in the irradiation system described by Tesei et al. [39].

### Measurement of cytosolic free calcium concentration

P2X7R activity as a Ca^2+^ channel was monitored by measuring changes in the cytosolic Ca^2+^ concentration using the fluorescent Ca^2+^ indicator Fura-2- acetoxymethyl ester (Fura-2/AM) (Thermo Fisher Scientific, cat n. F1201) as previously described [40]. Briefly, 10^6^ GBM cells were incubated with Fura-2/AM (4 μM) for 20 min at 37 °C in saline solution (125 mM NaCl, 5 mM KCl, 1 mM MgSO_4_, 1 mM NaH_2_PO_4_, 20 mM HEPES, 5.5 mM glucose, and 1 mM CaCl_2_ at pH 7.4) supplemented with sulfinpyrazone (250 μM). Ca^2+^ changes were measured at the excitation wavelength couple 340/380 nm, with emission set at 505 nm, in a thermostat-controlled (37 °C) and magnetically stirred Cary Eclipse Fluorescence Spectrophotometer (Agilent Technologies, Milano, Italy) after stimulation with different concentrations (100, 300, and 500 μM) of BzATP (Sigma-Aldrich, cat n. B6396). Pretreatment with AZ10606120 dihydrochloride (1 μM) (Tocris Bioscience, cat n. 3323/10) was used to selectively block P2X7R activity. Ca^2+^ concentration [Ca^2+^]i levels were calculated according to the general formula: [Ca^2+^]i = Kd (F − Fmin)/(Fmax − F) as previously described [41].

### Ethidium bromide uptake

Changes in plasma membrane permeability were measured by monitoring ethidium bromide uptake. About 10^6^ GBM cells were incubated at 37 °C in a thermostat-controlled and magnetically stirred cuvette of a Cary Eclipse Fluorescence Spectrophotometer (Agilent Technologies, Milano, Italy) in the presence of 20 μM ethidium bromide (Sigma-Aldrich) and exposed to different concentrations (100, 300, and 500 μM) of BzATP. Pretreatment with AZ10606120 dihydrochloride (1 μM) was used to selectively block the P2X7R. Fluorescence was measured at 360 nm excitation and 580 nm emission wavelength.

### Measurement of lactate dehydrogenase (LDH) in cell supernatants

Lactic dehydrogenase release was measured in the GBM culture supernatants using the LDH-Glo^TM^ Cytotoxicity Assay (Promega, cat n. J2380), according to the manufacturer’s instructions. Briefly, 1.5 × 10^5^/well GBM cells were plated in six-well plates. At the following time points from radiation treatment (1 day, 7 days, 14 days, 21 days, and 28 days), cell supernatants diluted 1:25 in LDH storage buffer (200 mM Tris-HCl (pH 7.3), 10% Glycerol, 1% BSA in deionized water) were added to LDH detection reagent at 1:1 ratio and incubated at room temperature for 60 min. Luminescence was measured using the GloMax^®^ bioluminescent reader (Promega). Percent LDH release (%), was calculated with the formula: LDH release (%) = [(experimental LDH release value) − (background value)]/[(LDH release value in 10% Triton X-100-treated samples) − (background value)] × 100. Lactic dehydrogenase release was normalized on the total amount of protein (µg).

### Annexin-V assay

Cell death induced by ATP treatment was assessed in a flow cytometer by Annexin-V-FITC Apoptosis Detection Kit (eBioscience™, Thermo Fisher Scientific, cat n. BMS500FI-300) as previously described by Zamagni et al. [42]. Immediately before flow cytometric analysis, propidium iodide (PI) was added to a final concentration of 5 µg/ml to discriminate total apoptotic cells (Annexin-V positive/PI negative and PI positive) from necrotic cells (Annexin-V negative/PI positive).

### Immunophenotypic analysis

GBM cells were analyzed for common stem cell markers by flow cytometry. Briefly, cells were collected, washed twice with PBS 1x, and stained with APC-conjugated anti-CD44 antibody (dilution 1:10) (Becton Dickinson, BD Pharmigen, cat n. 559942 RRID: AB_398683) and PE-conjugated anti- CD24 antibody (dilution 1:20) (Becton Dickinson, BD Pharmigen, cat n. 555428, RRID: AB_395822) for 30 min at 4 °C. APC-conjugated anti- CD133 antibody (dilution 1:10) (Miltenyi Biotec, cat n. 130-090-826) was used for single staining. After three washes, fluorescence was acquired using a FACS Canto flow cytometer (Becton Dickinson, San Diego, CA, USA) equipped with 488 and 633 laser lines. Appropriate isotype control was included for each sample.

### PCAWG and GTEx datasets analysis

Expression levels of the *P2RX7* gene were evaluated in specimens from healthy and tumor brain tissue data from PCAWG [43] and GTEx datasets. We screened 41 gliomas (17 oligodendroglioma and 24 glioblastomas) and 41 healthy brain specimens matched by sex and age. The RNA-seq data were downloaded from the ICGC data portal (https://dcc.icgc.org/) and from the GTEx portal (https://gtexportal.org/home/). Healthy brain tissues were also taken from the GTEX database according to Hardy scale of death classification selecting only class 2 death (Fast death for natural causes).

### Exon-sequencing analysis

Genomic DNA from eight GBM primary cell lines was extracted using the QIAamp DNA Mini kit (QIAGEN) according to the manufacturer’s instructions. DNA for each sample was quantified by means of Qubit 2.0 Fluorometer (Invitrogen), and subjected to quality control using the Agilent 2100 Bioanalyzer (Agilent Technologies). Samples were sequenced using the SureSelect Human All Exon V6 60 Mb Kit (Agilent) on HiSeq4000 (Illumina) 2 × 150 bp according to the manufacturer’s recommendations. The raw data were analyzed with Workflow Enrichment of Illumina BaseSpace. Alignment was performed with Isaac and somatic variant calling was carried out with Pisces. The detected variants were annotated using ANNOVAR. Variants were further filtered by position (located into exons and introns within 120 bp from the exon-intron boundary), and depth (>50 reads).

### Alkaline comet assay

The alkaline comet assay was performed according to the manufacturer’s protocol (Comet assay, Trevigen, Gaithersburg, MD). Tail DNA was analyzed with the CometAnalyzer tool as previously described by our laboratory [44], and expressed as described by Garcia et al. [45].

### Statistics

Data were shown as mean ± standard error of the mean (SEM). Data were analyzed and significance were calculated with GraphPad Prism 9.1 software (GraphPad, La Jolla, California, USA), with an unpaired two-tailed Student’s *t*-test. PCAWG datasets were analyzed applying the nonparametric Wilcoxon test. Digital PCR data were analyzed applying Mann–Whitney nonparametric test. *P* values coding: n.s. not significant, *P ≤ 0.05; **P ≤ 0.01; ***P ≤ 0.001; ****P ≤ 0.0001. Supplementary materials and methods are available at Cell Death and Disease website.

## Results

### The P2X7R is expressed in patient-derived GBM cells with stemness features

**Fig. 1:**
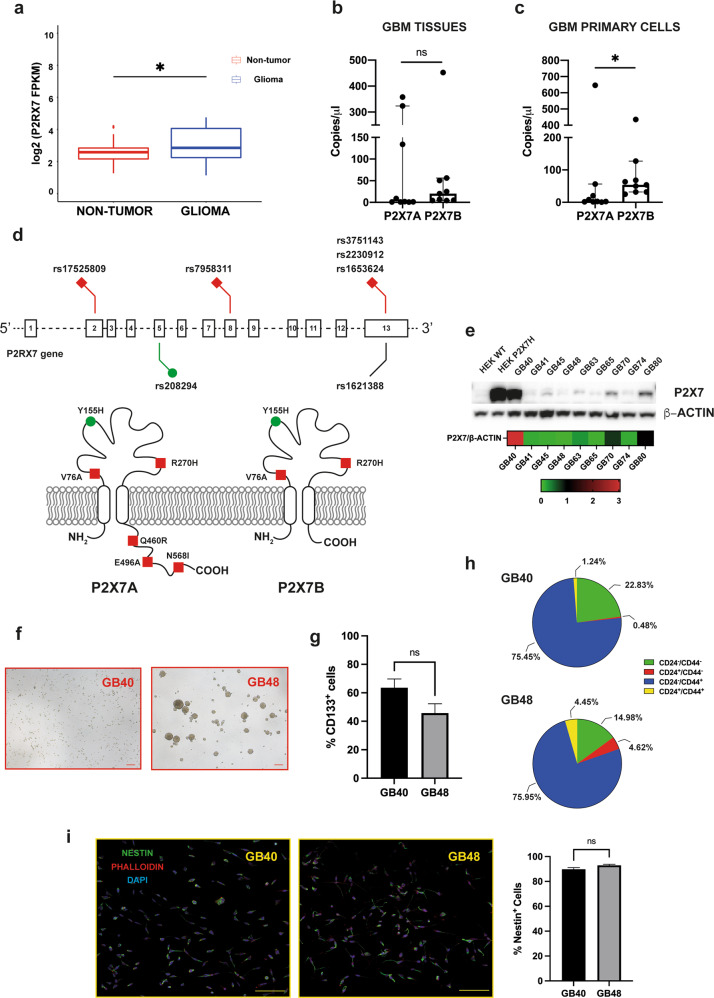
P2X7R is expressed in glioma. **a** Expression of P2RX7 in the healthy brain (red) and glioma (oligodendroglioma and glioblastoma) (blue) based on PCAWG and GTEx data. Statistical analyses were conducted with the Wilcoxon test. **b, c** Digital PCR of P2RX7A and P2RX7B expression from nine GBM specimens (**b**) and the nine derived GBM primary cell lines (**c**). Data are expressed as the median of transcript copies in 1 µl with 95% CI. Statistical analyses were conducted with Mann–Whitney test. **d** Schematic representation of *P2RX7* gene structure and exonic genetic variants found in patient-derived GBM primary cells. Schematic representation of the membrane topology of P2X7R isoforms A and B reporting position and amino acid substitution of major SNPs. Red, loss-of-function SNPs; green, gain-of-function SNPs. **e** Western blot showing full-length P2X7R (P2X7A) expression in patient-derived GBM primary cells. β-ACTIN was used to normalize P2X7R expression. Heatmap shows P2X7R densitometric values normalized on β-actin expression. Wild-type HEK293 (HEK293-WT) and HEK293 cells transfected to express human full-length P2X7R (HEK293-P2X7) were used as negative and positive control, respectively. **f** Brightfield images showing the morphology of GB40 and GB48 primary cells. Scale bar = 200 μm. **g** CD133 immunophenotype of GB40 and GB48 cells expressed as a percent of positive cells. Data are shown as mean ± SEM, *n* = 3. Statistical analyses were conducted with unpaired two-tailed Student’s *t*-test. **h** Representative CD24 and CD44 immunophenotype of GB40 and GB48 cells expressed as a percent of positive cells for each combination of the two markers. **i** Confocal images of nestin expression in GB40 and GB48 cell lines. Green = nestin-positive cells; Red = phalloidin positive cells; Blue = DAPI nuclei staining. Images were taken with a Nikon A1R confocal microscope at 20 X magnification. Scale bar = 100 μm. Data are shown as mean ± SEM; *n* = 5. Statistical analyses were conducted with unpaired two-tailed Student’s *t*-test. *P* values: n.s not significant; *P ≤ 0.05.

We initially verified P2X7R expression in a PCAWG dataset [43]. P2X7R expression was significantly higher in glioma samples (17 oligodendrogliomas and 24 glioblastomas) compared to the healthy brain (*p* = 0.03) (Fig. 1a). Whether the P2X7R is functional in this dataset is not known, nor expression of the B isoform was reported. To clarify this issue, we investigated the expression of the P2X7A and B isoforms in nine GBM native tissues (Supplementary Table S1) and in the primary cell lines thereof derived (Fig. 1b, c). In native GBM tissues, the B isoform was expressed to a higher level compared to isoform A, but the difference was not statistically significant (Fig. 1b). On the contrary, the B versus A isoform was significantly (*p* = 0.0188) overexpressed in the cell lines established from the native tumors (Fig. 1c). We then performed whole-exome sequencing on the GBM-derived primary cell lines to verify the presence of SNPs. Seven exonic and nine intronic SNPs were identified in four GBM samples (Table 1 and Supplementary Table S2, respectively). Among the exonic SNPs, all four GBM samples shared the loss-of-function 1729T > A (rs1653624) SNP that changes an asparagine with isoleucine (N568I) in the C-terminal domain thus impairing P2X7R translocation to the plasma membrane (Fig. 1d), and the gain-of-function 489 C > T (rs208294) SNP (H155Y) (Table 1). Two primary cell lines (GB48 and GB63) also exhibited the 1513A > C (rs3751143) loss-of-function SNP (Table 1). To verify if P2X7R expression was affected by these SNPs, protein levels were evaluated by Western blot with an antibody recognizing both the P2X7A and P2X7B variants (Fig. 1e). P2X7R expression was highly variable in GBM primary cell lines, with the GB40 cells expressing the highest and GB48 the lowest level (Fig. 1e and Supplementary Fig. S1a). To further investigate the P2X7R function, we focused on the two GBM cell lines expressing the highest and the lowest P2X7R levels. As GBM is a highly heterogeneous tumor, with a rich stem cell population, we also evaluated growth profile, multipotency potential, and stemness marker expression.

**Table 1:** Exonic variants of P2RX7 gene in GBM cells.

GBM ID	Variant	Variant effect	Localization	Annotation	Variant allele frequency	Protein change	Effect on P2X7 function
GB40	c.T463C	nonsynonymous SNV	Exon 5	rs208294	43.2%	Y155H	Gain
	c.G809A	nonsynonymous SNV	Exon 8	rs7958311	5.9%	R270H	Loss
	c.A1379G	nonsynonymous SNV	Exon 13	rs2230912	5.6%	Q460R	Loss
	c.A1703T	nonsynonymous SNV	Exon 13	rs1653624	5.3%	N568I	Loss
	c.G1746A	synonymous SNV	Exon 13	rs1621388	3.9%	P582P	Neutral
GB48	c.T463C	nonsynonymous SNV	Exon 5	rs208294	32.3%	Y155H	Gain
	c.A1487C	nonsynonymous SNV	Exon 13	rs3751143	49.3%	E496A	Loss
	c.A1703T	nonsynonymous SNV	Exon 13	rs1653624	100%	N568I	Loss
GB63	c.T463C	nonsynonymous SNV	Exon 5	rs208294	61.9%	Y155H	Gain
	c.A1487C	nonsynonymous SNV	Exon 13	rs3751143	64.3%	E496A	Loss
	c.A1703T	nonsynonymous SNV	Exon 13	rs1653624	8.6%	N568I	Loss
GB70	c.T227C	nonsynonymous SNV	Exon 2	rs17525809	9.5%	V76A	Loss
	c.T463C	nonsynonymous SNV	Exon 5	rs208294	100%	Y155H	Gain
	c.A1379G	nonsynonymous SNV	Exon 13	rs2230912	8.7%	Q460R	Loss
	c.A1703T	nonsynonymous SNV	Exon 13	rs1653624	4.8%	N568I	Loss
	c.G1746A	synonymous SNV	Exon 13	rs1621388	6.4%	P582P	Neutral

GB40 grows as single cells or in small aggregates, while GB48 cells form small neurospheres (Fig. 1f). In addition, GB48 showed a faster growth rate compared to GB40 (Supplementary Fig. S1b). Both GB40 and GB48 showed a similar percentage of cells positive for the stemness markers CD133 (63.57 and 45.77%, respectively) (Fig. 1g) and nestin (89.26 and 90.94%, respectively) (Fig. 1i). Co-immunophenotyping with CD24^+^ and CD44^+^ antibodies showed a predominant population of CD44^+^-only positive cells in both GB40 and GB48 (75.45 and 75.95% respectively) (Fig. 1h), while GB48 cells showed a small population enriched for the CD24^+^ marker in both CD44^+^ and CD44^−^ cells (Fig. 1h, lower pie chart). GBM cells differentiated as previously described [38] to test their multipotency upregulated both astroglial (GFAP) and oligodendroglial (GalC) markers (Supplementary Fig. S1c, d). Altogether, these data show that patient-derived cell lines retain the typical GBM traits.

### P2X7R is functional in patient-derived primary GBM cell lines

**Fig. 2:**
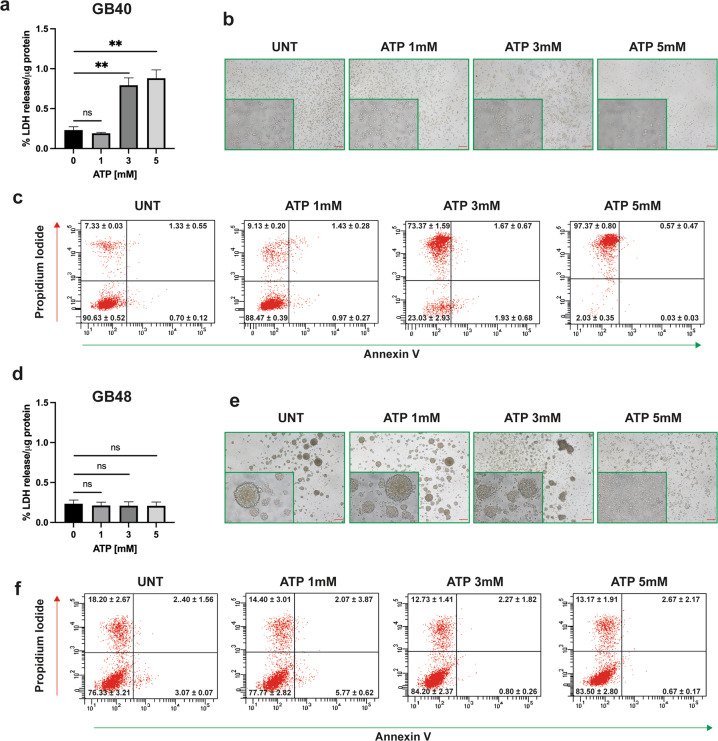
ATP induces cell death in GBM primary cell lines. **a**, **d** ATP-dependent cytotoxicity in GBM cell lines measured as percent LDH release normalized on the total amount of protein (µg). Data are shown as mean ± SEM; *n* = 4. Statistical analyses were conducted with unpaired two-tailed Student’s *t*-test. **b, e** Brightfield images showing GB40 and GB48 cell morphology after 24 h of incubation with ATP. Scale bars = 200 μm for largest images and 20 μm for insets. **c, f** ATP-dependent Annexin-V staining. *P* values: n.s not significant; **P ≤ 0.01.

**Fig. 3:**
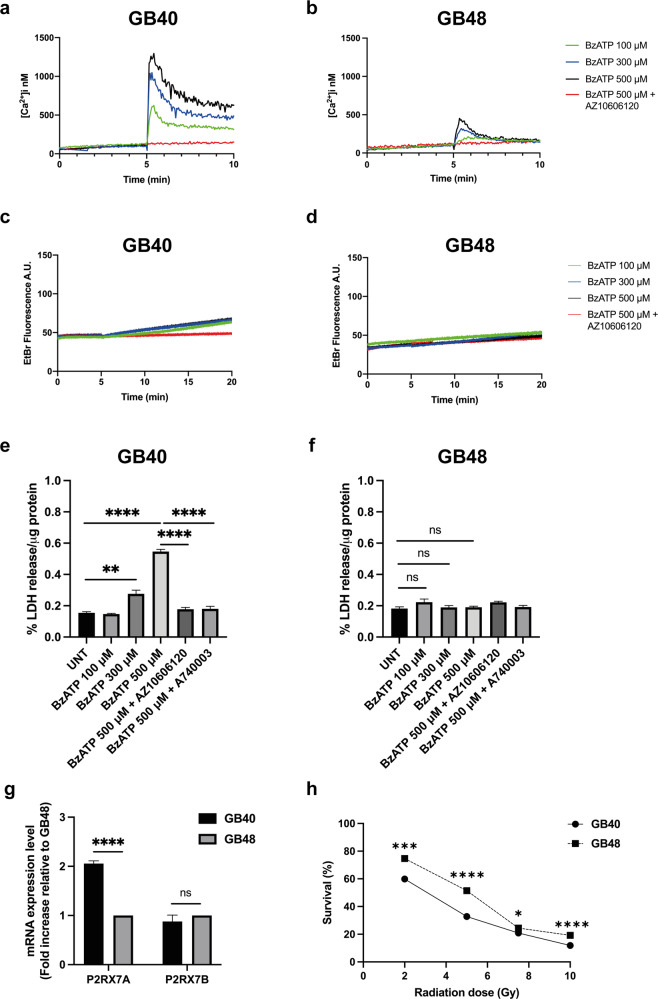
Differential P2X7R-dependent responses in GBM primary cell lines. **a**, **b** Representative traces showing BzATP -dependent cytosolic Ca^2+^ increase in the absence or presence of 5 min pretreatment with AZ10606120 (1 μM). **c**, **d** Representative traces showing ethidium bromide uptake following stimulation with increasing concentrations of BzATP alone or applied after 5 min pretreatment with AZ10606120 (1 μM). **e**, **f** BzATP-dependent cytotoxicity in GBM cell lines in the absence or presence of P2X7R antagonists AZ10606120 or A740003, measured as percent LDH release normalized to the total amount of protein (µg). Data are shown as mean ± SEM; *n* = 3. Statistical analyses were conducted with unpaired two-tailed Student’s *t*-test. **g** mRNA expression levels of P2X7A and P2X7B isoforms in GB40 and GB48 cells. Data were normalized to HPRT-1 and GAPDH housekeeping genes. Data are shown as mean ± SEM; *n* = 3. Statistical analyses were conducted with unpaired two-tailed Student’s *t*-test. **h** Percentage of surviving cells measured by soft agar assay at 28 days from radiation treatment at different doses in GB40 and GB48. Data are shown as the mean ± SEM; *n* = 6. Statistical analyses were conducted with unpaired two-tailed Student’s *t*-test. *P* values: n.s not significant; *P ≤ 0.05; **P ≤ 0.01; ***P ≤ 0.001; ****P ≤ 0.0001.

GB40 and GB48 cells exhibited strikingly different responses to stimulation with eATP. Prolonged (24 h) incubation in the presence of increasing eATP concentrations triggered extensive cell death in GB40 but not in GB48 cells (Fig. 2a, b, d). These data were confirmed by Annexin-V/propidium iodide (PI) assay, where a large amount of dead GB40 cells (predominantly necrotic Annexin-V^−^/PI^+^ cells) was observed following treatment with high eATP doses (Fig. 2c, f and Supplementary Fig. S2a, b). Interestingly in GB48 cells, exposure to high eATP doses that were not per se cytotoxic, caused dramatic morphological changes characterized by the disaggregation of the neurospheres into single cells (Fig. 2e). To dissect the differential responses to eATP, we investigated the activity of P2X7R as an ion channel and as a macropore by measuring intracellular Ca^2+^ changes (Fig. 3a, b) and plasma membrane permeability increases by ethidium bromide uptake (Fig. 3c, d). ATP analog BzATP was used as a semi-selective P2X7R agonist. In line with P2X7R expression levels, maximal Ca^2+^ increase in GB40 was much (about three fold) higher, and more prolonged compared to GB48 cells, showing a kinetic typical of macropore opening (Fig. 3a). The Ca^2+^ increase was fully obliterated in both cell lines by the selective negative allosteric modulator AZ10606120 (Fig. 3a, b, red line). GB40 but not GB48 cells showed a clear BzATP-stimulated ethidium bromide uptake which was fully obliterated by AZ10606120 (Fig. 3c, d). Finally, like eATP, also BzATP was a potent cytotoxic stimulus in GB40 but not in GB48 cells (Fig. 3e, f). BzATP-induced cytotoxicity was inhibited by antagonist treatment (Fig. 3e, f). Altogether these data show that GB40 and GB48 differentially express the P2X7R, which in turn confers differential sensitivity to eATP. Indeed, GB40 cells express higher levels of A isoform than GB48, instead the expression level of the B isoform is similar between the two cell lines (Fig. 3g). Such differences in P2X7R expression and function also affected response to irradiation, with GB40 being more sensitive than GB48 (Fig. 3h); this is in line with previous data reporting P2X7R as a good prognostic indicator of radiosensitivity [37].

### Radiation induces cell death and ATP release in GBM cells

To verify how and if radiotherapy affects purinergic signaling in GBM in vitro, we mimicked an accelerated hypofractionated regimen by treating GB40 and GB48 cells with a 7.5 Gy radiation dose. Cells were then followed for 28 days post-irradiation (p.i.), and periodically checked (at days 1, 7, 14, 21, and 28) to monitor morphological changes, lactic dehydrogenase release, and eATP release, P2X7A and B levels, and expression of senescence markers.

**Fig. 4:**
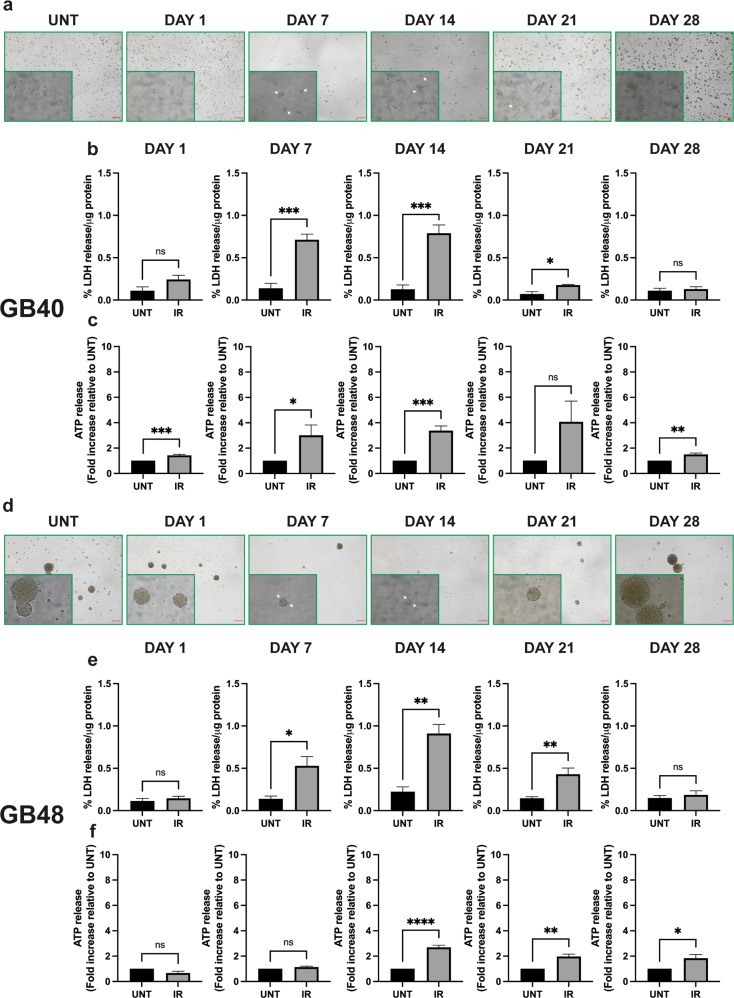
Radiation triggers cell death and ATP release in GBM cells. **a**, **d** Brightfield images of GB40 and GB48 cells at increasing intervals after radiation treatment with 7.5 Gy dose. Scale bars = 200 μm for largest images and 20 μm for insets. The white arrow indicates swollen GBM cells. **b**, **e** Radiation-induced percent LDH release normalized to the total amount of protein (µg). Data are shown as mean ± SEM; *n* = 4. Statistical analyses were conducted with unpaired two-tailed Student’s *t*-test. **c**, **f** Radiation-induced ATP release measured in the culture supernatants. Data were normalized on the total amount of protein (µg) and reported as a relative increase in eATP released by irradiated cells compared to untreated cells at each time point. Data are shown as mean ± SEM, *n* = 5. Statistical analyses were conducted with unpaired two-tailed Student’s *t*-test. *P* values: n.s not significant; *P ≤ 0.05; **P ≤ 0.01; ***P ≤ 0.001; ****P ≤ 0.0001.

Representative pictures of GB40 and GB48 cells at different time points after 7.5 Gy dose are shown in Fig. 4a, d. Cell death peaked at p.i. day 14 in both GBM cell lines, to slowly decline at later time points (Fig. 4b, e). GB40 cells were more susceptible to early (p.i. day 1) radiation damage than GB48 cells. At p.i. day 7, diffuse morphological changes (cell shrinkage and plasma membrane ruffles) compatible with dying cells were also observed (Fig. 4a, d). At p.i. day 21, both cell lines started to recover from irradiation and repopulated the flasks, although cell death was still higher in GB48 cells compared to untreated cells (*p* = 0.009). Moreover, resistant GB40 cells proliferated preferentially as small aggregates from p.i day 21 (Fig. 4a). At p.i. day 28, GB40 and GB48 cell death declined to control levels, suggesting that both cell lines had fully recovered from irradiation (Fig. 4b, e). Irradiation caused a large eATP release in the GB40 cell line at all time points (Fig. 4c). Instead, eATP released from GB48 cells increased starting from p.i day 14 (Fig. 4f). Irradiation thus causes a long-lasting stimulation of eATP release that prolongs beyond the acute phase of radiation-induced cell death.

### Senescent phenotype in GBM cells surviving radiation-induced DNA damage

**Fig. 5:**
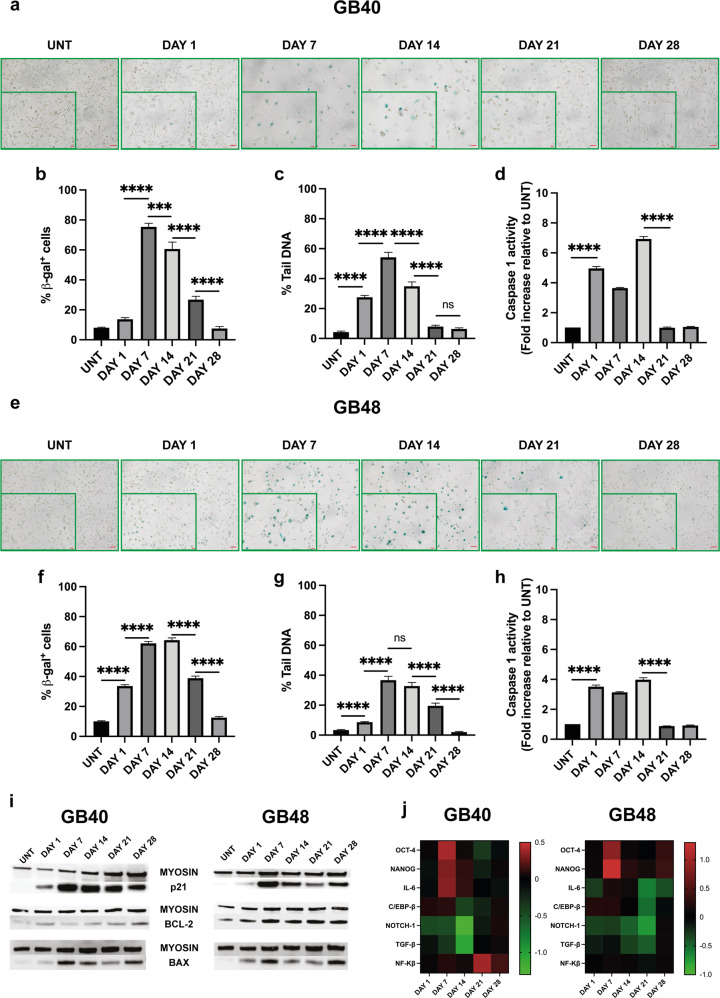
Radiation induces a senescent phenotype and extensive DNA damage. **a**, **e** β-Galactosidase staining of GB40 and GB48 cells at increasing intervals after radiation treatment. Scale bars = 50 µm. A zoomed area is shown for each time point, scale bars = 20 µm. **b**, **f** Percent of β-galactosidase positive cells expressed as the mean ± SEM, *n* = 5. **c**, **g** DNA damage expressed as percent tail DNA. Data are reported as mean ± SEM, *n* > 150. **d**, **h** Caspase-1 activity at increasing intervals after radiation treatment. Data are reported as a fold increase in caspase-1 activity of irradiated cells compared to untreated cells at each time point. Data are shown as mean ± SEM; *n* = 3. **i** Western blot analysis of p21, Bax, and Bcl-2 expression at increasing intervals after radiation treatment. Myosin was used as housekeeping. **j** Heatmap shows mRNA expression levels of OCT-4, NANOG, IL-6, C/EBP-β, NF-Kβ, NOTCH-1, and TGF-β in GB40 and GB48 at increasing intervals after radiation treatment. Data were normalized to HPRT-1 and GAPDH housekeeping genes. Data are reported as log_10_ of the mean, *n* = 3. All statistical analyses were conducted with unpaired two-tailed Student’s *t*-test. *P* values: n.s not significant, ***P ≤ 0.001; ****P ≤ 0.0001.

Irradiation of malignant cells usually causes cell death and permanent proliferative inactivation referred to as cellular senescence. Despite general consensus that senescence inhibits tumor progression, recent studies highlighted a potentially adverse long-term effect due not only to chronic inflammation associated with the senescence-associated secretory phenotype (SASP) but also to the resumption of proliferation in the senescent population of cancer cells [46, 47]. The P2X7R induces senescence in the immune system [48], thus we investigated whether there was a correlation between P2X7R expression and activity, and senescence in irradiated GB40 and GB48 cells. In GB40, β-galactosidase (β-gal) positive cells, a senescence marker, peaked at p.i. days 7 and 14 (Fig. 5a, b), and then declined to a pre-irradiation level at p.i. day 28. A similar trend occurred in GB48 cells, although β-gal positive cells were present as early as p.i. day 1 (Fig. 5e, f). Senescent cells are able to repair DNA during the 28 days p.i. irradiation time, as shown by the alkaline comet assay. Tail DNA (a readout of DNA damage) increased significantly at p.i. day 1, reaching the maximum at p.i. days 7 and 14, and decreasing thereafter (Fig. 5c). DNA damage trend was similar in both cell lines (Fig. 5g and Supplementary Fig. S3a–d) even if the percentage of tail DNA was significantly higher in GB40 (p.i. day 7 peak 54.24% vs 36.68%, for GB40 and GB48, respectively). Cellular senescence is associated with pyroptosis [49], thus we verified if caspase-1, a key cell death-associated, P2X7R-dependent, response is activated in irradiated GBM cells. A statistically significant increase in caspase-1 activity occurred in GB40 and GB48 cells at p.i. days 1, 7, and 14 (Fig. 5d, h). Compatible with the senescence trend, at p.i. days 21 and 28 caspase-1 activity decreased to levels comparable to untreated cells (Fig. 5d, h). As a further indication of senescence, p21 protein increased in both cell lines, peaking at p.i. day 7, and slowly declining thereafter (Fig. 5i). Bax and Bcl-2 levels increased in both cell lines throughout the experiment (Fig. 5i). These data show that radiation triggers senescence, extensive DNA damage, and pyroptotic cell death in a large fraction of GB40 and GB48 cells. Surviving cells replace the original population over a 28 days time window and regain a pre-irradiation phenotype. We then tested if radiation-induced senescence and stemness are linked evaluating the expression of OCT-4 and NANOG, stemness factors involved in the maintenance of stem cell identity, self-renewal, and pluripotency. At p.i. day 7, an increase in OCT-4 and NANOG expression was observed in both GB40 and GB48 (Fig. 5j and Supplementary Figs. S4, S5). This was paralleled by a significant decrease in NOTCH-1 and TGF-β expression at p.i. day 1, and a slight increase in C/EBP-β (Fig. 5j and Supplementary Figs. S4, S5), a senescence marker often associated with an increased risk of tumor development [47, 50].

### P2X7R variant shift is associated to post-irradiation cell recovery

**Fig. 6:**
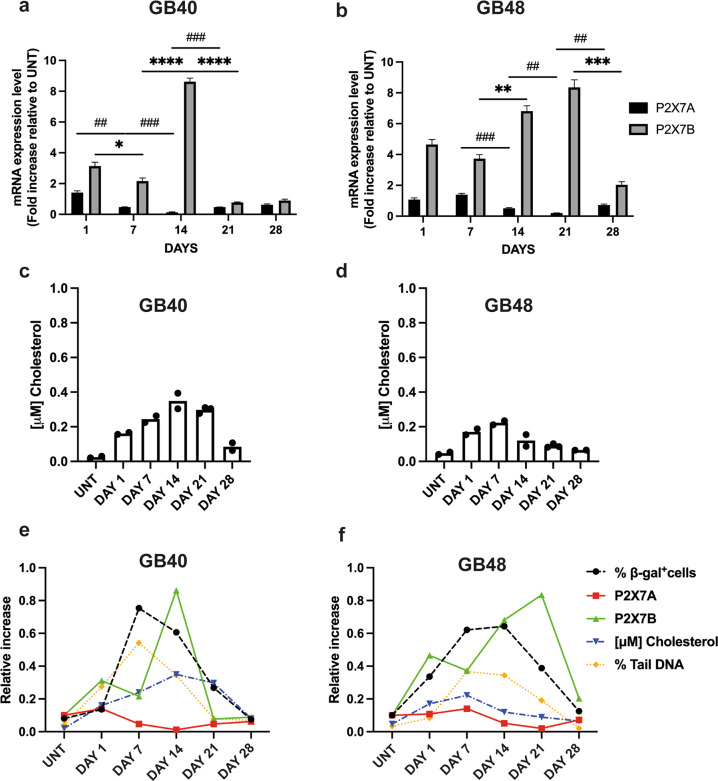
Changes in P2X7A and P2X7B isoform expression and cholesterol content in radiation-resistant GBM clones during recovery. **a** mRNA expression levels of P2X7A and P2X7B isoforms in GB40 cells at increasing intervals radiation treatment. Data were normalized to HPRT-1 and GAPDH housekeeping genes. Data are shown as mean ± SEM; *n* = 3. **b** mRNA expression levels of P2X7A and P2X7B isoforms in GB48 cells at increasing intervals after radiation treatment. Data were normalized to HPRT-1 and GAPDH housekeeping genes. Data are shown as mean ± SEM; *n* = 3. **c**, **d** Total cellular cholesterol content measured at increasing intervals after radiation treatment. Single data are reported, *n* = 2. **e**, **f** Correlation between P2X7A/B mRNA expression levels, DNA damage (expressed as percent tail DNA), senescence marker (expressed as percent β-gal positive cells), and the total amount of cholesterol at increasing intervals after radiation treatment. Statistical analyses were conducted with unpaired two-tailed Student’s *t*-test. *P* values: *P ≤ 0.05; **P ≤ 0.01; ***P ≤ 0.001; ****P ≤ 0.0001; ^##^*P* ≤ 0.01; ^###^*P* ≤ 0.001.

To verify if P2X7R variants are differentially modulated by radiation treatment, we analyzed expression levels of the P2X7A and B isoforms. P2X7A expression declined at p.i. days 7–21 and 14–21 in GB40 and GB48, respectively (Fig. 6a, b), while P2X7B underwent a several-fold increase peaking at p.i. days 14 and 21 in GB40 and GB48, respectively, to return at pre-irradiation levels at p.i. day 28 (Fig. 6a, b). Altogether, these data suggest that radiation promoted the selection of radiation-resistant GBM clones expressing a high level of the P2X7B isoform and low levels of the P2X7A isoform. A senescence-modulating factor is cholesterol [51, 52]. This lipid is of interest because it is also known to negatively modulate P2X7R function [22]. A striking increase in the total amount of cholesterol was observed in both cell lines at p.i. day 1 (Fig. 6c, d), reaching a maximum at p.i. day 14 (GB40) or 7 (GB48). At p.i. day 28 cholesterol returned to near pre-irradiation levels in both cell lines. Interestingly, β-gal positive cells, tail DNA accumulation, and P2X7B expression showed a similar time course, peaking slightly earlier in GB40 and later in GB48 cells. The cholesterol time course followed that of β-gal positive cells, tail DNA accumulation, and P2X7B expression in GB40 but not GB48 cells. P2X7A expression followed a quite different, and opposite time course, being almost abrogated at those time points (e.g., p.i. days 14 or 21) where β-gal positive cells, tail DNA accumulation, cholesterol level, and P2X7B expression were maximal (Fig. 6e, f).

**Fig. 7:**
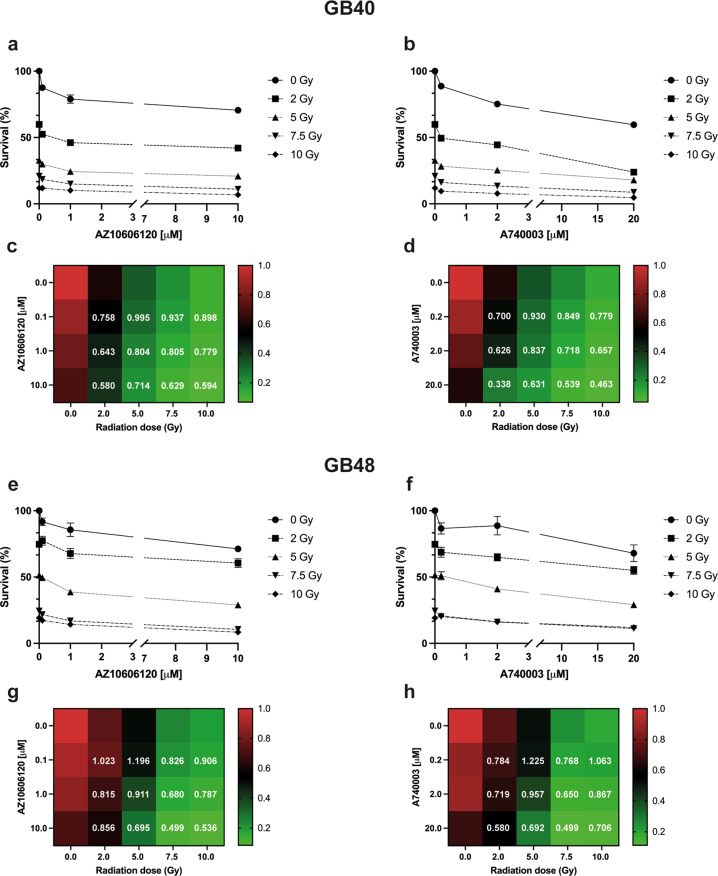
P2X7R antagonist’s treatment potentiates irradiation cytotoxic effects in GBM. Percentage of surviving cells measured by soft agar assay at 28 days from radiation treatment in the absence or presence of P2X7R blockade with AZ10606120 or A740003 in GB40 (**a**, **b**) and GB48 (**e**, **f**). Data are shown as the mean ± SEM; *n* = 6. Heatmap shows the number of colonies (survival fraction) measured by soft agar assay at 28 days from radiation treatment in the absence or presence of P2X7R blockade in GB40 (**c**, **d**) and GB48 (**g, h**). Data were shown as the mean; *n* = 6. Combination index (CI) analysis of GB40 and GB48 cells treated with increasing concentrations of irradiation, AZ10606120 and A740003 are reported in white. The additive, synergistic, and antagonistic effect of the combinations was calculated using Compusyn Software (CI <1 synergism; CI = 1 additivity; CI >1 antagonism).

The P2X7R has often been dubbed a dual-function (or Dr. Jekyll/Mr. Hyde) receptor [53] as while stimulation with high eATP concentrations precipitates cell death, stimulation with low doses promotes survival. There is ample in vivo evidence showing that P2X7R pharmacological blockade inhibits tumor progression. Irradiation causes a large eATP release, thus we wondered whether P2X7R blockade during p.i. recovery would reduce or rather increase radiation-induced GBM death. As shown in Fig. 7, treatment with two different P2X7R antagonists (AZ10606120 and A740003) during the recovery phase reduced survival in both GB40 and GB48 cells especially at 2 and 5 Gy in GB40 and GB48, respectively. In GB40, the Chou-Talalay analysis showed that the combination index (CI) values between AZ10606120 and different radiation doses ranged from 0.580 to 0.995 (Fig. 7c), while CI values between A740003 and radiation doses ranged from 0.338 to 0.930 (Fig. 7d), indicating in both cases a synergism between irradiation and administration of P2X7R antagonists. The strongest synergism was given by combination treatment with 2 Gy radiation dose and the highest doses of AZ10606120 (10 µM) or A740003 (20 µM) (Fig. 7c, d). In GB48, CI analysis showed a strong synergism at 7.5 Gy in combination with AZ10606120 at 10 µM dose or with A740003 at 20 µM (Fig. 7g, f). Finally, we observed antagonism for the combination of radiation with the lowest doses of AZ10606120 (0.1 µM) or A740003 (0.2 µM). The combination of radiotherapy with P2X7R-targeting drugs may be a more effective treatment than radiation alone.

## Discussion

Identification of new potential therapeutic targets is urgently needed in GBM. While there is little doubt that the P2X7R has an important role in tumor growth and host–tumor interaction [13, 54], its contribution to GBM growth and progression is as yet controversial. Contrasting observations from different laboratories may be due to differential expression in GBM and differential modulation in different experimental conditions of the two main human P2X7 variants, i.e., P2X7A and P2X7B.

The P2X7B isoform might be of special relevance in cancer because, on the one hand, it retains the growth-promoting ability, while on the other it does not support the well-known P2X7A-associated cytotoxicity [25]. Analysis of the PCAWG dataset showed that the P2X7R is expressed in glioma tissues, but screening of various GBM cell lines established from GBM patients revealed a wide variation in expression of the P2X7 protein, suggesting the presence of a large individual heterogeneity. Yet, screening of GBM patient-derived tissue samples and the matched primary GBM cell cultures by qPCR revealed higher expression of P2X7B mRNA versus P2X7A.

Differential expression of the P2X7A and B isoforms conferred a dramatically differential sensitivity to eATP-mediated cytotoxicity.

The high P2X7A-expressing GB40 cells were eminently susceptible, while the low P2X7A-expressing GB48 were refractory to eATP-dependent cytotoxicity, consistent with previous observations showing that P2X7A is a radiation-responsive gene and a positive prognostic factor for response to radiotherapy [37].

Hypofractionated irradiation with 7.5 Gy caused extensive pyroptosis, cell death, and DNA damage in both GB40 and GB48 cell lines, but GB40 cells were more susceptible than GB48. During the p.i. recovery phase, a cell subpopulation characterized by enhanced P2X7B and reduced P2X7A expression progressively emerged, emphasizing the contribution of P2X7B to cell proliferation and repair. During the p.i. phase GBM cells underwent extensive phenotypic changes characterized by upregulation of senescence and stemness markers, and overexpression of both the proapoptotic Bax and the antiapoptotic Bcl-2 gene, a likely indication of radiation-stimulated apoptosis on one hand, and of the apoptosis-refractory phenotype of the radiation-resistant cells on the other.

During p.i. recovery, both GB40 and GB48 accumulated cholesterol. This lipid, which accumulates during senescence is also a negative modulator of P2X7A-dependent macropore formation [22]. During the p.i. the most successful surviving cells are those which reduce the probability of macropore opening by increasing the cholesterol content and thus down modulating P2X7A.

Radiotherapy deeply impacts TME biochemical composition inducing an accumulation of DAMPs including eATP [11]. Extracellular ATP accumulation in the TME may have two opposite effects on the tumor cells: on one hand, it may accelerate tumor cell death by activating the P2X7A macropore, but on the other, it may provide a growth stimulus mainly acting at the P2X7B. The experiments reported in Fig. 7 show that a functional P2X7B receptor is needed during the p.i. recovery because incubation in the presence of P2X7R blockers during this phase reduces survival. Of course, the inhibitors used do not discriminate between P2X7A and P2X7B (no antagonists with these features are currently available), but being P2X7A strongly down-modulated, it is very likely that their effects are due to P2X7B blockade [32–36, 55].

We detected several *P2RX7* SNPs in four patients’ derived cells, namely loss-of-function 1729T > A and 1513A > C, which are rare in the general population. Increased frequency of loss-of-function SNPs may suggest that in GBM mutations are selected that decrease P2X7A-dependent response while leaving unaltered those mediated by P2X7B since both 1729T > A and 1513A > C are located on the carboxyl-terminal tail which is absent in P2X7B.

**Fig. 8:**
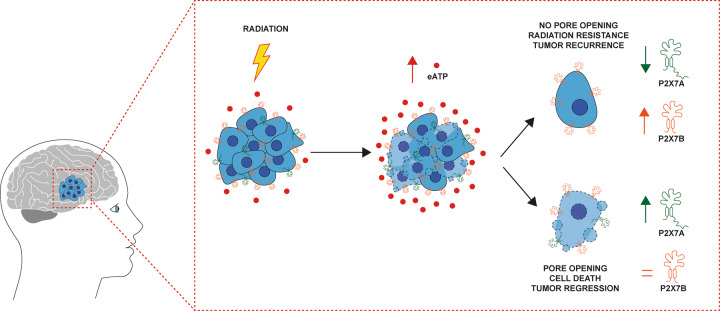
Radiation induces P2X7 isoforms switch in GBM. Radiotherapy induces cell death in glioblastoma (GBM) and triggers ATP release into the tumor microenvironment (TME). Irradiated GBM cells undergo a P2X7R isoform switch, with overexpression of the truncated P2X7B variant and the downregulation of the full-length P2X7A variant. These radiation-resistant clones are responsible for tumor recurrence.

Resistance to radiation treatment, mostly dependent on the activation of DNA repair [56–59] and senescence, is a crucial issue in GBM therapy. Senescence was initially described as a permanent inactivation of proliferation status that eventually results in cell death [60, 61], however, it is now clear that senescence-associated cell-cycle arrest does not necessarily leads to apoptosis. Cancer cells may exploit senescence for long-lasting survival in a quiescent status, allowing for DNA repair, metabolic activity rewiring, and proliferation resumption. This behavior suites well human GBM, a tumor known for its high plasticity and ability to adapt to a hostile microenvironment. We show here that in GBM cells senescence occurs in parallel with a shift in expression of P2X7R isoforms and pro- or antiapoptotic genes, and in overexpression of stemness transcription factors, suggesting a link between these survival-promoting responses. We hypothesize that radiotherapy promotes a dramatic P2X7R isoform switch (Fig. 8) relevant for the onset of resistance and provide a rationale for P2X7R-targeting in the post-irradiation phase.

## Supplementary information

Supplementary material

NPJ checklist

## Data Availability

The datasets used during the current study are available from the corresponding authors on reasonable request and after authorization from the Institute.
