# Supplementary material for: Irradiation causes senescence, ATP release, and P2X7 receptor isoform switch in glioblastoma
Source: Cell Death Dis. 2022 Jan 24;13(1):80. doi: 10.1038/s41419-022-04526-0 (PMC8786947; doi:10.1038/s41419-022-04526-0)
Supplement: Supplementary file 1 — Supplementary material [file 41419_2022_4526_MOESM1_ESM.docx]

**Supplementary Appendix**

**Irradiation causes senescence, ATP release, and P2X7 receptor isoform switch in glioblastoma**

Michele Zanoni^1#^, Alba Clara Sarti^2^, Alice Zamagni^1^, Michela Cortesi^1^, Sara Pignatta^1^, Chiara Arienti^1^, Michela Tebaldi^3^, Anna Sarnelli^4^, Antonino Romeo^5^, Daniela Bartolini^6^, Luigino Tosatto^7^, Elena Adinolfi^2^, Anna Tesei^1*#^, Francesco Di Virgilio ^2*#^.

^1^Biosciences Laboratory, IRCCS Istituto Romagnolo per lo Studio dei Tumori (IRST) "Dino Amadori" Meldola, Italy.

^2^Department of Medical Sciences, Section of Experimental Medicine, University of Ferrara, Ferrara, Italy.

^3^Biostatistics and Clinical Trials Unit, IRCCS Istituto Romagnolo per lo Studio dei Tumori (IRST) "Dino Amadori", Meldola, Italy.

^4^Medical Physics Unit, IRCCS Istituto Romagnolo per lo Studio dei Tumori (IRST) "Dino Amadori", Meldola, Italy.

^5^Radiotherapy Unit, IRCCS Istituto Romagnolo per lo Studio dei Tumori (IRST) "Dino Amadori", Meldola, Italy.

^6^Pathology Unit, M. Bufalini Hospital, AUSL Romagna, Cesena, Italy.

^7^Department of Neurosurgery, M. Bufalini Hospital, AUSL Romagna, Cesena, Italy.

^*^These authors contribute equally to this work.

^#^ To whom correspondence should be addressed:

Biosciences Laboratory, IRCCS Istituto Romagnolo per lo Studio dei Tumori (IRST) "Dino Amadori", Via Piero Maroncelli 40, 47014, Meldola, Italy. Phone: +39 0543739996, +39 0543739932, emails: [michele.zanoni@irst.emr.it](mailto:michele.zanoni@irst.emr.it); [anna.tesei@irst.emr.it](mailto:anna.tesei@irst.emr.it).

Department of Medical Sciences, Section of Experimental Medicine, University of Ferrara, Via Luigi Borsari 46, 44121, Ferrara, Italy. Phone: +39 0532455353, email: [fdv@unife.it](mailto:fdv@unife.it).

**Supplementary methods**

**RNA extraction and Real-time Quantitative Polymerase Chain Reaction**

Total RNA extraction and quantitative Real-time PCR were performed as previously described ^1^, and expression of P2RX7A, P2RX7B isoforms and POU5F1, NANOG, NFKB1, IL6, CEBPB, NOTCH1 and TGFB1 genes was assessed. The amount of mRNA was normalized to the endogenous genes GAPDH and HPRT-1.

**Digital PCR**

Digital PCR (dPCR) was used to evaluate the expression level of P2RX7A and P2RX7B isoforms in nine GBM tissues and matched primary cells using the chip based QuantStudioTM 3D Digital PCR system (Applied Biosystems, Foster City, CA, USA). Ten ng of P2RX7A and P2RX7B cDNA were used for amplification. Reaction mixes containing either cDNA or water (no-template controls) were first prepared by adding 2X QuantStudio 3D™ Digital PCR Master Mix v2 (Thermo Fisher Scientific, cat n. A26358) and 20X gene specific assay in a total volume of 15.5 μl. Chips were run using GeneAmp PCR System 9700 (Applied Biosystems, Foster City, CA, USA) under the following conditions: hold at 96°C for 10 min; 45 cycles of 60°C for 2 min and 98°C for 30 sec; hold at 60°C for 2 min. At the end of the reaction, chips were processed using the QuantStudioTM 3D Digital PCR system (Applied Biosystems) and analyzed with QuantStudioTM 3D Analysis SuiteTM software (version 3.0.3).

**Soft agar assay combination indexes analysis**

GBM cells (10^3^ per well) were mixed with 0.4% Seaplaque agar in growth medium with antibiotics, plated on top of a solidified layer of 1% Agar Noble in HBSS medium supplemented with antibiotics in a 24-well plate. Six samples were prepared for each treatment dose. Freshly prepared growth medium, with or without P2X7R-targeting agents, was added every 2 days. Colonies with more than 50 cells were weekly quantified under inverted microscope (Olympus IX51 microscope, Olympus Corporation, Tokyo, Japan) by two independent observers. Data, expressed as survival fraction, are reported after 28 days from radiation treatment. Drug combination indexes were evaluated according to the Chou-Talalay equation ^2^, using Compusyn Software (ComboSyn Incorporated, Paramus, NJ, USA) as previously described ^3^. Based on developer instructions we defined: synergism where CI < 1; additivity where CI = 1; antagonism where CI > 1.

**Measurement of extracellular ATP levels**

ATP levels were measured in the culture supernatants with ENLITEN rLuciferase/Luciferin reagent (Promega, cat n. FF2021), according to manufacturer’s instructions. Briefly, 1.5x10^5^/well of GBM cells were plated in six-well plates. At the following time points after radiation treatment (1 day, 7 days, 14 days, 21 days and 28 days) luminescence was measured with the GloMax® bioluminescent reader (Promega) immediately after the addition of 50μl of ENLITEN reagent to 50 μl of cell supernatant. Data are normalized on total amount of protein (µg) and reported as relative increase over untreated controls.

**Measurement of total cholesterol**

Total amount of cellular cholesterol was quantified using the Amplex Red cholesterol assay kit (Invitrogen™, Thermo Fisher Scientific, cat n. A12216), according to the manufacturer's instructions. Briefly, 2.5x10^5^ GBM cells were collected at the following time points after radiation treatment (1 day, 7 days, 14 days, 21 days and 28 days), snap freezed in liquid nitrogen and resuspended in 50 μl of 1X Reaction Buffer working solution. One μg of total protein was used in the assay for each sample. Fluorescence was measured in a Perkin Elmer Wallac Victor3 1420 system (Perkin Elmer, Wellesley, Massachusetts, USA) according to manufacturer’s instructions.

**Western blot**

Cells were detached and lysed in RIPA lysis buffer (Santa Cruz, cat n. sc-24948) for 1h at 4°C. Equal protein amounts were separated on Novex NuPage Bis-Tris 4–12% precast gel (Life Technologies, Thermo Fisher Scientific) and transferred to nitrocellulose membranes (GE Healthcare-Life Sciences, Milano, Italy). Membranes were blocked in TBS–Tween-20 (0.1%) supplemented with 5% non-fat powdered milk for 1 h to saturate unspecific binding sites. Membranes were then incubated overnight with primary antibodies at 4 °C. The anti-Bcl2 polyclonal antibody (NeoMarkers, Fremont, CA, cat n. MS-123-P) (dilution 1:200), the anti-Bax antibody (Cell signaling technology, cat n. 2772) (dilution 1:1000) and the anti-p21 antibody (Cell signaling technology, clone 12D1, cat n. 2947) (dilution 1:800) were incubated in TBS–Tween-20 (0.1%) supplemented with 5% non-fat powdered milk. The anti-myosin (Sigma-Aldrich, cat n. M8064) was diluted 1:1000. The anti-P2X7R polyclonal antibody (Merck-Millipore, cat n. AB5246) was diluted 1:1000 and incubated in TBS–Tween-20 (0.1%) supplemented with 2% of BSA. Membranes were incubated with secondary goat anti-rabbit HRP-conjugated antibodies (Invitrogen™, Thermo Fisher Scientific, cat n. 31460) at a 1:3,000 dilution for 1 h at room temperature. Protein bands were visualized by CYANAGEN ECL HRP Chemiluminescent Substrate Reagent Kit ETA C ULTRA 2.0 (Cyanagen Srl, Bologna, Italy) with a Licor C-Digit Model 3600.

**β-Galactosidase assay**

Senescent cells were visualized through β-Galactosidase staining performed with the Senescence β-Galactosidase Staining Kit (Cell Signaling, cat n. 9860), according to the manufacturer's instructions. Briefly, GBM cells were plated at a concentration of 1.5x10^5^/well in 6 well plate pre-coated with Matrigel diluted 1:100 in HBSS medium supplemented with antibiotics in order to force cells attachment at each time point (1 day, 7 days, 14 days, 21 days and 28 days). Cells were then fixed in 1X fixative solution for 10 minutes at room temperature and stained overnight at 37°C with the β-galactosidase staining solution at pH 6.0. Images were acquired with an inverted Olympus IX51 microscope (Olympus Corporation, Tokyo, Japan) with a 10X objective, equipped with a Nikon Digital Sight DS-Vi1 camera (CCD vision sensor, square pixels of 4.4 μM side length, 1600 × 1200-pixel resolution, 8-bit grey level) (Nikon Instruments). Percentage of β-Galactosidase positive cells were reported.

**Inflammasome Caspase-1 activity assay**

Caspase 1 activity was measured using the Caspase-Glo 1 inflammasome assay kit (Promega, cat n. G9951), according to manufacturer's instructions.One hundred microliters of substrate solution was added to each well of a 96-well plate at each time point, plates were mixed for 30 s and then incubated at room temperature for 2 hours to allow stabilization of the luminescent signal. Luminescence was measured using the GloMax® bioluminescent reader (Promega). Data are expressed as relative increase over untreated controls.

**Immunofluorescence**

GBM cells were plated at 3x10^4^/well on a glass slide pre-coated with Matrigel diluted 1:100 in HBSS medium supplemented with antibiotics in order to force cells attachment. Cell were then fixed in paraformaldehyde 4% for 15 minutes at room temperature, washed three times in PBS 1X and blocked for 1 hour in blocking solution (1X PBS/5% normal goat serum/0.3% Triton X-100) and then incubated overnight at 4°C with primary anti-Nestin antibody (Merck Millipore, clone 10C2, cat n. MAB5326) at dilution 1:200. Differentiated GBM cells were stained overnight at 4°C with primary anti-Galactocerebroside antibody (Sigma Aldrich, cat n.G9152) or primary anti-Glial Fibrillary Acidic Protein (Sigma Aldrich, cat. n G9269) at 1:50 and 1:100 dilution respectively. After washing in PBS 1X, slides were incubated 1 hour at room temperature with secondary goat anti-mouse or anti-rabbit Alexa Fluor™ 488 (Invitrogen™, cat n. A-11001 and A-11008 respectively) at 1:300 dilution. Slides were then washed 5 times with PBS 1X stained with Alexa Fluor™ 594 Phalloidin (Invitrogen™, Thermo Fisher Scientific, cat n. A12381) at dilution 1:40. Slides were then stained with DAPI and mounted with ProLong™ Diamond Antifade Mountant (Invitrogen™, Thermo Fisher Scientific, cat n. P36961). The confocal imaging was performed with a Nikon A1 confocal laser scanning microscope, equipped with a 20X objective (0.75 NA), using 405, 488 and 561 nm laser lines.

**Supplementary data**

**Table S1. GBM patients’ characteristics.** n/a not applicable. MGMT O-6-methylguanine-DNA Methyltransferase. TMZ temozolomide.

| Patient Characteristics |  |  |  |
| --- | --- | --- | --- |
| Median age at diagnosis (min/max) |  | 70 | 41/84 |
|  |  | n | % |
| Gender | male | 7 | 77.8 |
|  | female | 2 | 22.2 |
|  |  |  |  |
| Tumor site | Frontal | 3 | 33.3 |
|  | Parietal | 1 | 11.1 |
|  | Temporal | 5 | 55.6 |
|  |  |  |  |
| MGMT-status | unmethylated | 0 | 0.0 |
|  | methylated | 8 | 88.9 |
|  | n/a | 1 | 11.1 |
|  |  |  |  |
| Extent of resection | Subtotal | 3 | 33.3 |
|  | Gross | 4 | 44.5 |
|  | n/a | 2 | 22.2 |
|  |  |  |  |
| Chemotherapy (TMZ) after surgery | yes | 7 | 77.8 |
|  | no | 1 | 11.1 |
|  | n/a | 1 | 11.1 |
|  |  |  |  |
| Radiotherapy after surgery | yes | 7 | 77.8 |
|  | no | 1 | 11.1 |
|  | n/a | 1 | 11.1 |

**Table S2. Intronic variants of P2RX7 gene in GBM primary cells.**

| **GBM ID** | **Variant** | **Variant Effect** | **Localization** | **Annotation** | **Variant allele frequency** | **Protein change** | **Effect on P2X7 function** |
| --- | --- | --- | --- | --- | --- | --- | --- |
| **GB40** | **c.615-5C>G** | **splicing** | **Intron 6** | **rs208307** | **47.1%** | **-** | **?** |
|  | **c.615-116T>G** | **-** | **Intron 6** | **rs208306** | **56.4%** | **-** | **?** |
|  | **c.615-21_615-18delTTTG** | **-** | **Intron 6** | **rs36144485** | **6.3%** | **-** | **?** |
|  | **c.745-102C>T** | **-** | **Intron 7** | **rs504677** | **99.1%** | **-** | **?** |
|  | **c.1290+19dupT** | **-** | **Intron 12** | **-** | **12.5%** | **-** | **?** |
|  | **c.1290+34delT** | **-** | **Intron 12** | **rs112146943** | **7.5%** | **-** | **?** |
| **GB48** | **c.1290+19dupT** | **-** | **Intron 12** | **-** | **10.8%** | **-** | **?** |
| **GB63** | **c.1290+19dupT** | **-** | **Intron 12** | **-** | **41.6%** | **-** | **?** |
|  | **c.1290+34delT** | **-** | **Intron 12** | **rs112146943** | **50.5%** | **-** | **?** |
| **GB70** | **c.615-5C>G** | **splicing** | **Intron 6** | **rs208307** | **15,1%** | **-** | **?** |
|  | **c.437-47C>T** | **-** | **Intron 4** | **rs208293** | **100%** | **-** | **?** |
|  | **c.615-116T>G** | **-** | **Intron 6** | **rs208306** | **100%** | **-** | **?** |
|  | **c.615-21_615-18delTTTG** | **-** | **Intron 6** | **rs36144485** | **100%** | **-** | **?** |
|  | **c.745-102C>T** | **-** | **Intron 7** | **rs504677** | **14.3%** | **-** | **?** |
|  | **c.973-27G>A** | **-** | **Intron 9** | **rs73220273** | **4.4%** | **-** | **?** |
|  | **c.1290+34delT** | **-** | **Intron 12** | **rs112146943** | **3.6%** | **-** | **?** |
|  | **c.1290+33_1290+34delTT** | **-** | **Intron 12** | **-** | **6.7%** | **-** | **?** |

**Supplementary Figure S1.**

**
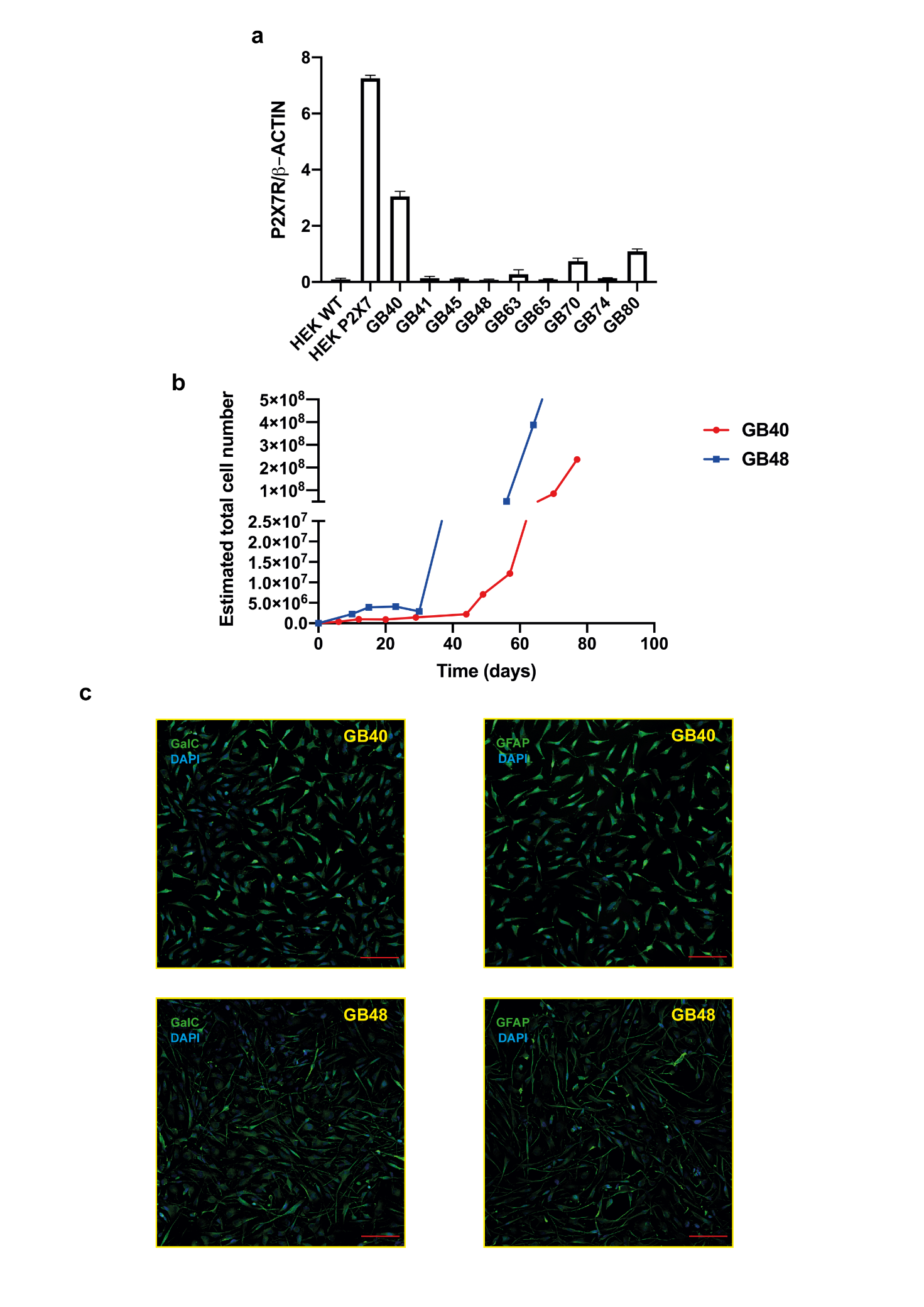
**

**Supplementary Figure S1. a** Histograms shown the statistical analysis of densitometric values of P2X7R normalized on β-ACTIN expression. Data are represented as the mean ± SEM; n=3. **b** Rates of expansion of both GB40 (red line) and GB48 (blue line) cells. Cells counts were plotted into the y-axis while the days on the x-axis. **c** GB40 and GB48 primary cells differentiation show up regulation of astroglial (GFAP) and oligodendroglial (GalC) markers. Images are obtained with Nikon A1R confocal microscope. Green = GFAP and GalC positive cells; Blue = DAPI nuclei staining. Magnification 20x. Scale bar = 100 µm.

**Supplementary Figure S2.**

**
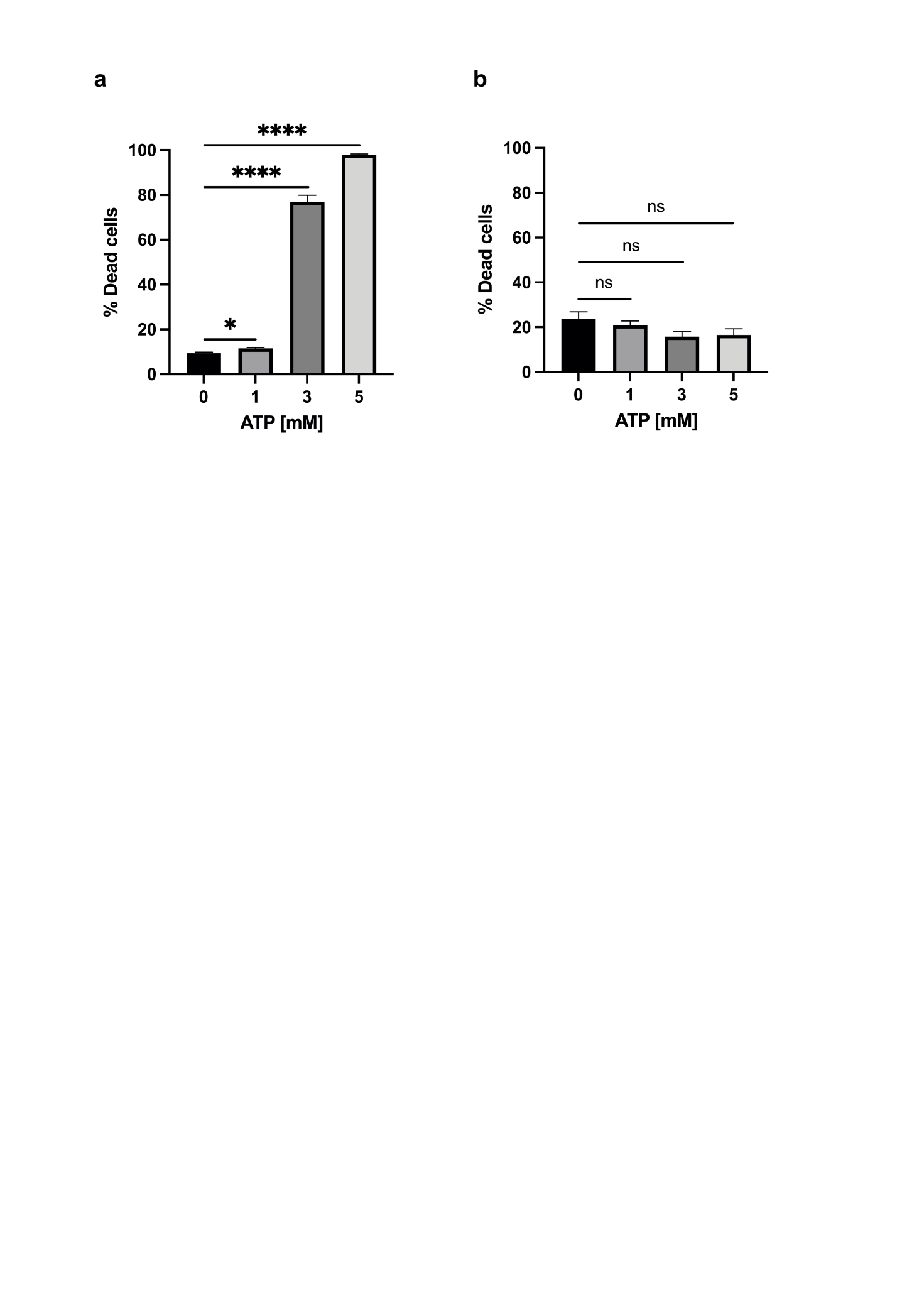
**

**Supplementary Figure S2. Prolonged ATP exposure induces cell death in GB40 cells but not in GB48 cells. a-b** Percentage of dead cells (comprising Annexin-V+/PI- , Annexin-V+/PI+, Annexin-V+/PI+ cells) in GBM cells after ATP treatment (1, 3 and 5 mM) for 24h. Data are represented as the mean ± SEM; n=3. Statistical analyses were conducted with unpaired two-tailed Student’s t test. P values: n.s not significant; * P≤0.05; ****P≤0.0001.

**Supplementary Figure S3.**

**
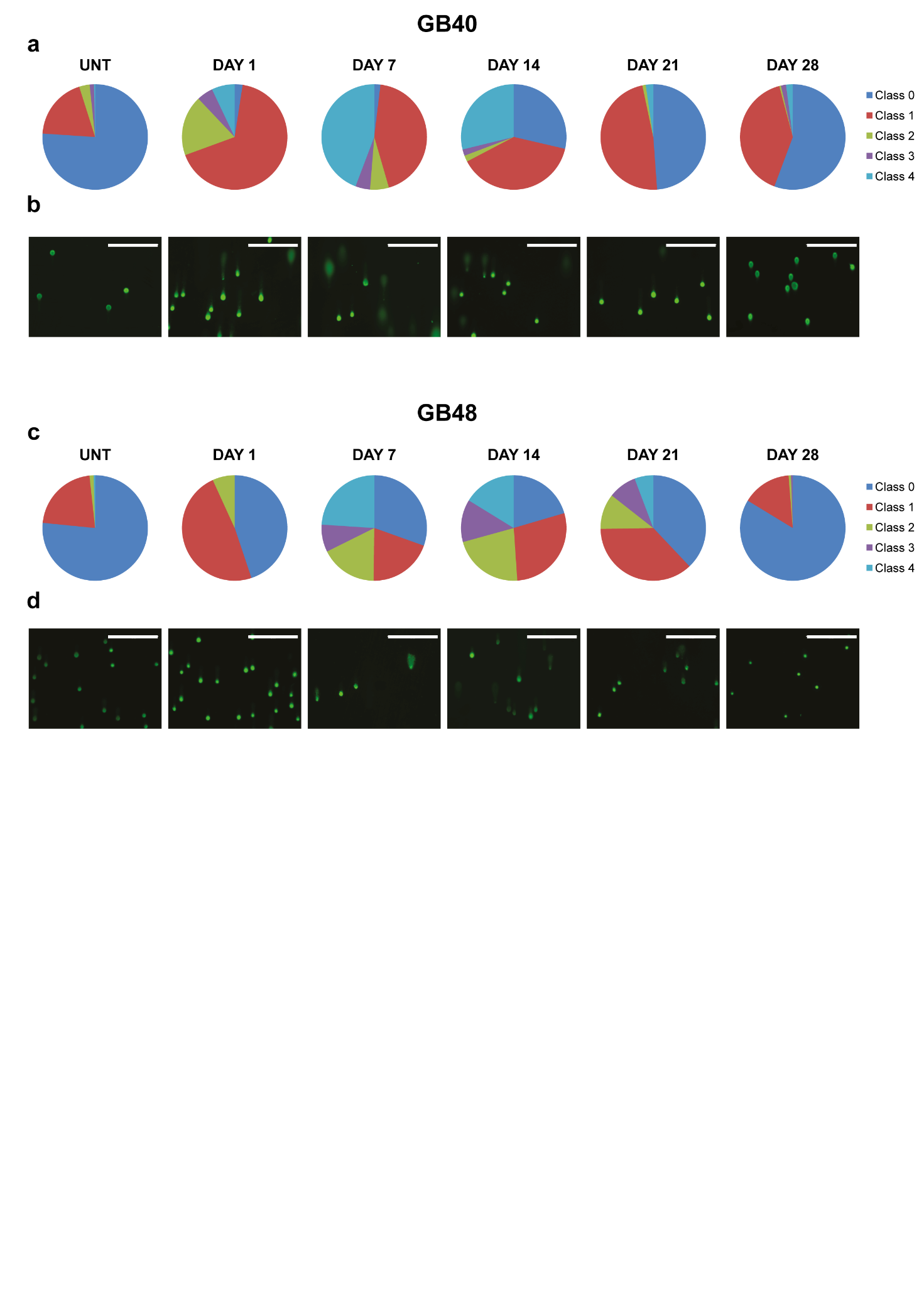
**

**Supplementary Figure S3. GBM cells recover from radiation-induced DNA damage. a, c** Pie chart graphs representative of comet classes distribution observed after 1, 7, 14, 21 and 28 days from radiation treatment in GB40 and GB48 cells respectively. Comets were categorized into 5 classes, from grade 0 to 4 according to % of tail DNA. **b, d** Representative photomicrographs of comet assay observed after 1, 7, 14, 21 and 28 days from radiation treatment in GB40 and GB48 cells respectively. DNA is stained with SYBR® Green. Magnification 10x. Scale bar = 400 µm.

**Supplementary Figure S4.**

**
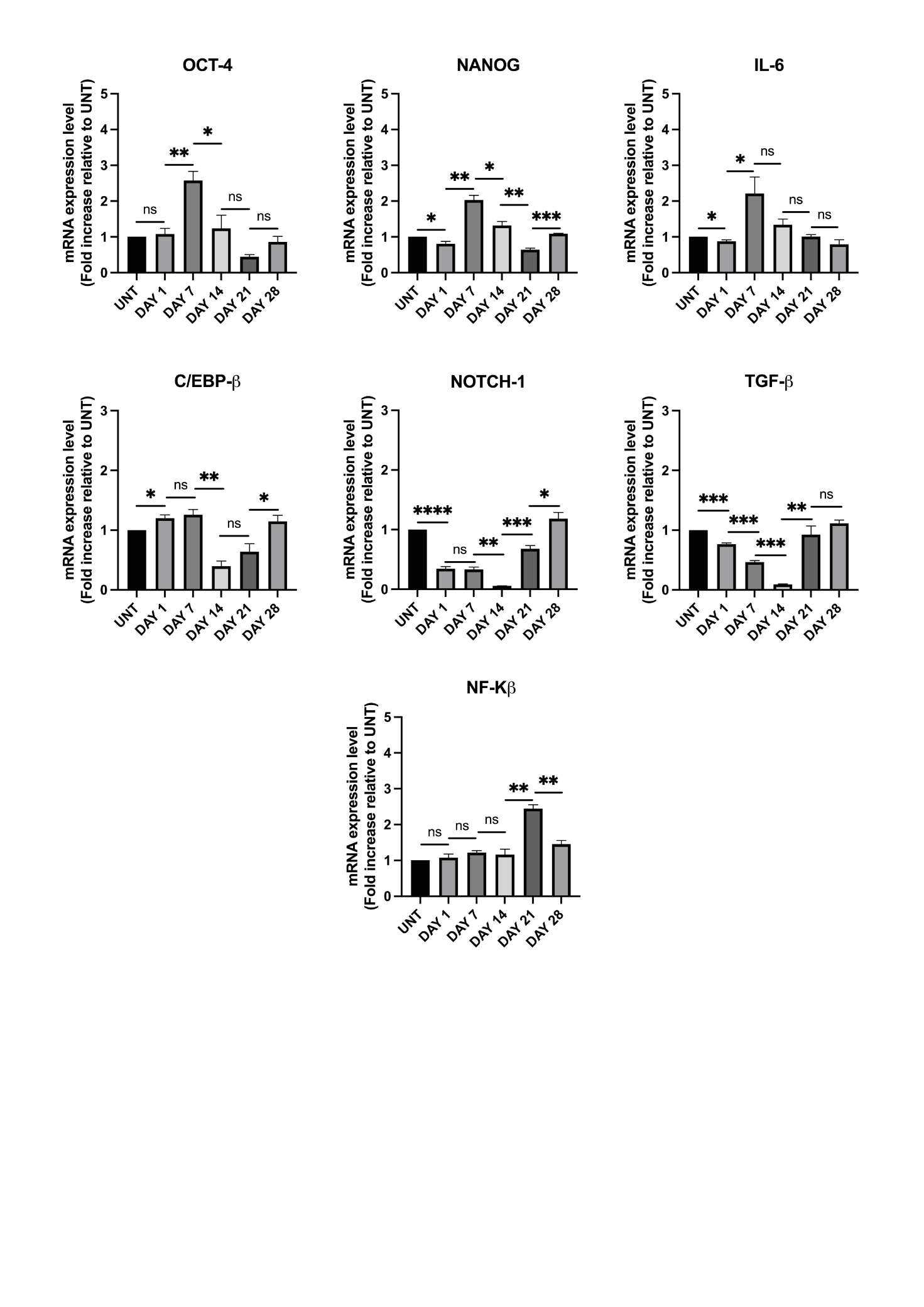
**

**Supplementary Figure S4. Radiation induces senescent and stemness phenotypes in GB40 cells.** mRNA expression levels of OCT-4, NANOG, IL-6, C/EBP-β, NOTCH-1, TGF-β, and NF-Kβ in GB40 cells at increasing intervals radiation treatment. Data were normalized to HPRT-1 and GAPDH housekeeping genes. Data are shown as mean ± SEM; n=3. Statistical analyses were conducted with unpaired two-tailed Student’s t test. P values: n.s, not significant; * P≤0.05; ** P≤0.01; ***P≤0.001; ****P≤0.0001.

**Supplementary Figure S5.**

**Supplementary Figure S5. Radiation induces senescent and stemness phenotypes in GB48. cells.** mRNA expression levels of OCT-4, NANOG, IL-6, C/EBP-β, NOTCH-1, TGF-β, and NF-Kβ in GB48 cells at increasing intervals radiation treatment. Data were normalized to HPRT-1 and GAPDH housekeeping genes. Data are shown as mean ± SEM; n=3. Statistical analyses were conducted with unpaired two-tailed Student’s t test. P values: n.s, not significant; * P≤0.05; ** P≤0.01; ***P≤0.001; ****P≤0.0001.


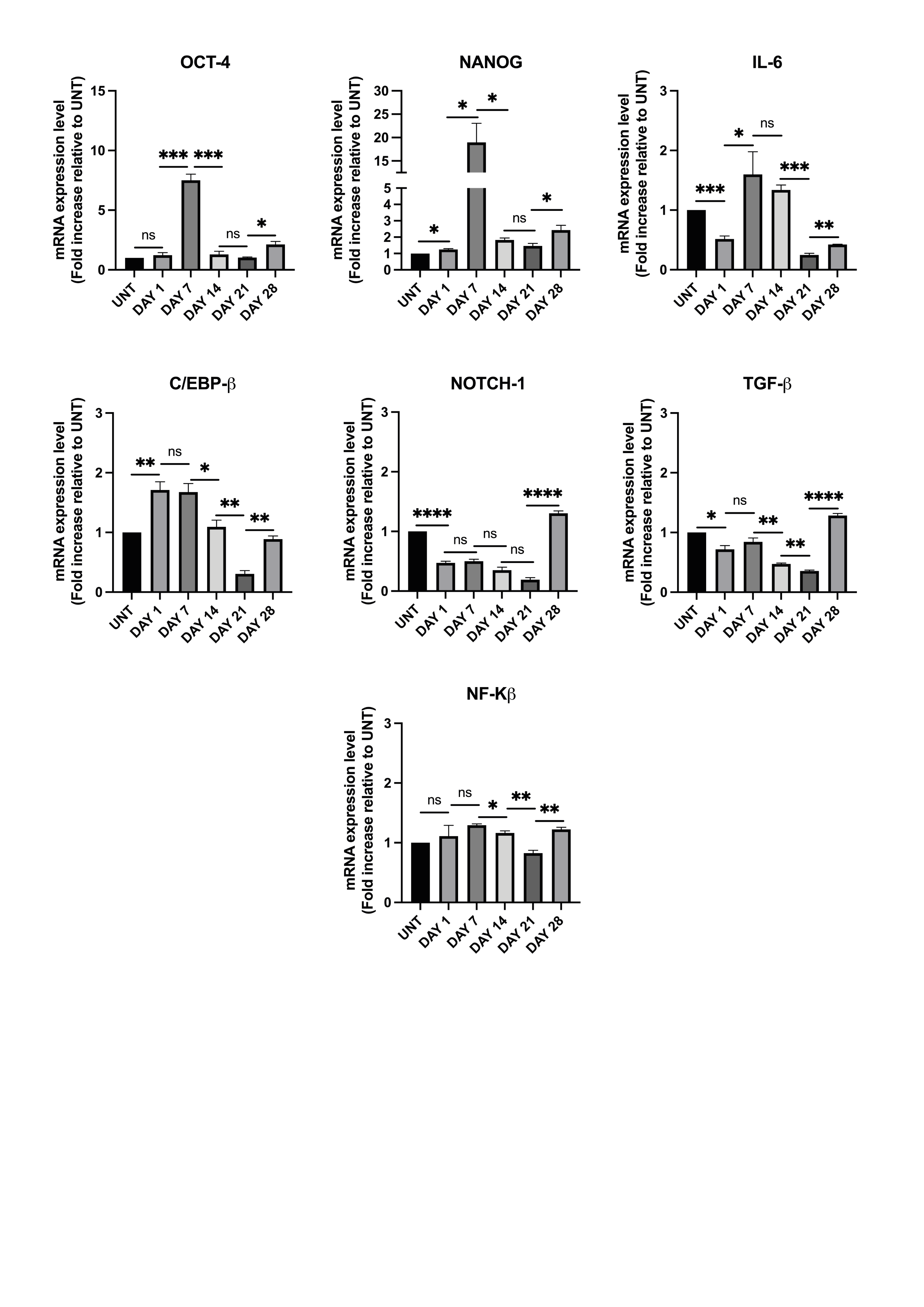


**References**

1 Tesei A, Cortesi M, Pignatta S, Arienti C, Massimo Dondio G, Bigogno C *et al.* Anti-tumor efficacy assessment of the sigma receptor pan modulator RC-106. A promising therapeutic tool for pancreatic cancer. *Front Pharmacol* 2019; **10**. doi:10.3389/fphar.2019.00490.

2 Chou TC. Drug combination studies and their synergy quantification using the chou-talalay method. Cancer Res. 2010; **70**: 440–446.

3 Di Rorà AGL, Bocconcelli M, Ferrari A, Terragna C, Bruno S, Imbrogno E *et al.* Synergism through WEE1 and CHK1 inhibition in acute lymphoblastic leukemia. *Cancers (Basel)* 2019; **11**. doi:10.3390/cancers11111654.
